# Proteomic Analysis of the Differential Protein Expression Reveals Nuclear GAPDH in Activated T Lymphocytes

**DOI:** 10.1371/journal.pone.0006322

**Published:** 2009-07-21

**Authors:** Wei-Yun Sheng, Tzu-Chien V. Wang

**Affiliations:** Department of Molecular and Cellular Biology, Chang Gung University, Kwei-San, Taiwan; Karolinska Institutet, Sweden

## Abstract

Despite the important role of T cell activation in the adaptive immunity, very little is known about the functions of proteins that are differentially expressed in the activated T cells. In this study, we have employed proteomic approach to study the differentially expressed proteins in activated T cells. A total of 25 proteins was characterized that displayed a decreased expression, while a total of 20 proteins was characterized that displayed an increased expression in the activated T cells. Among them, glyceraldehyde-3-phosphate dehydrogenase (GAPDH) was identified unexpectedly as one of the up-regulated proteins. Western blot analysis of proteins separated by 2-dimensional gel electrophoresis had identified several modified GAPDHs which were detectable only in the activated T cells, but not in resting T cells. These modified GAPDHs had higher molecular mass and more basic PI, and were present in the nucleus of activated T cells. Promoter occupancy studies by chromatin immunoprecipitation assay revealed that nuclear GAPDH could be detected in the promoter of genes that were up-regulated during T cell activation, but not in the promoter of genes that were not unaffected or down-regulated. Our results suggest that nuclear GAPDH may function as transcriptional regulator in activated T cells.

## Introduction

T lymphocytes, a key player in adaptive immunity, regulate all immune responses by interacting with antigen and by secreting cytokines. In a typical T cell immune response, the antigen presented by major histocompatibility complex is recognized by the T cell receptor to activate T cells [1]. Two major events occur in T cell activation, one is T cell proliferation (also called clonal expansion) to increase the number of antigen-specific T cells and the other is T cell differentiation, which transforms activated T cells into either memory cells or effectors.

T cell activation is known to mediate through complex signaling pathways that result in the expression and/or down-regulation of genes which are important for the physiological functions of the activated T cells [1]–[3]. Despite the important role of T cell activation in the adaptive immunity, very little is known concerning about the functions of proteins that are differentially expressed in the activated T cells. Two dimensional electrophoresis (2-DE) and mass spectrometry have been successfully used to analyze the protein expression pattern of lymphocytes, e.g., proteomic map of lymphoblastoid proteins [4], differentiation patterns of human Th1 and Th2 cells [5] and anti-CD3/anti-CD28 antibody-activated T helper cells [6]. As yet, none of these studies has attempted to examine the proteins that are differentially expressed in the activated T cells. In this study, we employed proteomic approach to investigate the proteins that are differentially expressed in the activated T cells.

## Methods

### Cell culture

The use of peripheral blood to cultivate T lymphocytes was reviewed and approved by institutional review board and ethics committee of Chang Gung Memorial Hospital. Informed written consent was obtained from healthy blood donors at Hsin-Chu Blood Centre, Taiwan. The peripheral blood lymphocytes (PBL) were isolated and cultured as previously described [7]. Activation of T cells was done by cultivating PBL in the presence of phytohemagglutinin (PHA) at 5 µg/ml.

### Two-dimensional electrophoresis (2-DE)

Proteins were extracted from cultured cells with a lysis solution containing 8 M urea, 4% CHAPS, 40 mM Tris-HCL, pH 8.0 and 1 mM PMSF, and were stored at −80°C prior to use. Separation of proteins in the first dimension was performed using the IPGphor™IEF system (Amersham Pharmacia Biotech). Briefly, 150 µg of proteins was mixed with 180 µl of rehydration solution containing 8 M urea, 2% CHAPS, 0.5% IPG buffer (pI 4–7 or 6–10 from Amersham Pharmacia Biotech.) and trace amounts of bromophenol blue. The mixtures were then applied onto 13 cm Immobiline Dry Strips (pI 4–7 or pI 6–10). After rehydrating the strips at 30 V, 12 h, the strips were electro- focused for 0.5 h at 50 V, 0.5 h at 100 V, 0.5 h at 250 V, 0.5 h at 500 V, 0.5 h at 1000 V, 0.5 h at 4000 V and 8 h at 8000 V. After focusing, the strips were first equilibrated in an equilibration buffer (6 M urea, 30% glycerol, 2% SDS, 50 mM Tris-HCl, pH 8.8) containing 65 mM DTT, and then in an equilibration buffer containing 53 mM iodoacetamide. For second dimensional separation, the equilibrated strips were placed onto 10% or 12% polyacrylamide gel containing 0.2% SDS and electrophoresed at 20 mA/gel for 4 h. The proteins in the gel were identified by silver staining [8].

### Analysis of proteins by MALDI-TOF MS

Protein spots of interest were excised from the gel separated by 2-DE, and washed with 25 mM NH_4_HCO_3_ containing 50% acetonitril for 15 min. The excised gels were next destained by a solution that contains 30 mM potassium ferricyanide and 200 mM sodium thiosulfate, and washed with 25 mM NH_4_HCO_3_. The gels were dehydrated by 100% acetonitril and dried. The proteins in the dried gel were digested with trypsin (20 µg/ml) for 8 h at 37°C. The digested peptide fragments were isolated by sonication for 10 min in 40% acetonitril that contains 0.4% trifluroacetic acid. For the identification of protein by matrix-assisted laser desorption/ionization-time of flight mass spectrometry (MALDI-TOF MS) and/or TOF/TOF, the peptide spectra of protein were calibrated against standard peptides derived from angiotensin II and adrenocorticotropic hormone (ACTH) and the database search was performed with Mascot (http://www.matrixscience.com) program using NCBI and SWISS-PROT databases.

### Western blot

Analysis of proteins by Western blot was essentially the same as previously described [9]. The anti-GAPDH was obtained from Abcam Limited, Cambridgeshire, UK.

### RNA extraction and RT-PCR

Total RNA from cells was extracted by TRIzol isolateion reagent (Invitrogen) following manufacturer's instruction. The conditions for RT-PCR were essentially the same as previously described [7]. The sequences of the primers used in the RT-PCR for the identification of various genes were listed in Table 1.

**Table 1:** The sequence of primers used in RT-PCR and ChIP.

Gene	RT-PCR primers	ChIP primers
hTERT	*LT5*: 5′-CGGAAGACTGTCTGGAGCAA	*PF*: 5′-TGCCCCTTCACCTTCCAG
	*LT6*: 5′-GGATGAAGCGGA GTCTGG A	*PR*: 5′-CAGCGCTGCCTGAAACTC
cMyc	*F*: 5′-AGAGTCTGGATCACCTTCTGCTGG	*PF*: 5′-TCGAGAAGGGCAGGGCTT
	*R*: 5′-ACGGACAGGATGTATGCTGTGG	*PR*: 5′-TGCCTCTCGCTGGAATTA
GAPDH	*WW144*: 5′-TGGTATCGTGGAA	*PF*: 5′-TACTAGCGGTTT
	GGACTCATGAC	TACGGGCG
	*WW145*: 5′-TGCCAGTGAG	*PR*: 5′-TCGAACAGGAGG
	CTTCCCGTTCAGC.	AGCAGAGAGCGA
B23	*F*: 5′-TTGTTGAAGCAGAGGCAATG	*PF*: 5′-AAGCATGGGCCTGCTTGTTG
	*R*: 5′-ACTTCCTCCACTGCCAGAGA	*PR*: 5′-GAGAGCTGCCATCACAGTAC
Cx3CR1	*F*: 5′-TGACTGGCAGATCCAGAGGTT	*PF*: 5′-ATGAGCTCTCCCAGCTTCCT
	*R*: 5′-GTAGAATATGGACAGGAACAC	*PR*: 5′-ATCTTTAGATGCTGCCACAGG
H2B	*F*: 5′-AGACGGCAAGAAGCGCAAGCG	*PF*:5′-GGATTTGCGAAT
	*R*: 5′- GCCAGGCGGGAAGCCTCACCT	CCTGATTGGGCA
		*PR*: 5′-AGCACTGTGTAG
		CTATAAAGCGCC
PKC β1	*F*: 5′-GACCATGGACCGCCTGTACTTTG	*PF*: 5′-ATCCCATTGGTCATTCTGCA
	*R*: 5′-GAAGAACAGACCGATGGCAAT	*PR*: 5′-GATCTACTGAAATCCTTCCTC
PKC δ	*F*: 5′-CTGCAAGAAGAACAATGGCAAG	*PF*: 5′-ATCCAAGGAATGGGCGAGCTC
	*R*: 5′-ATCCACGTCCTCCAGGAAATACT	*PR*: 5′-CCTCAGGGAAGCCTTTCCGAG
β-actin	*F*: 5′-TGTATGCCTCTGGTCGTACCAC	*PF*: 5′-CATTTAGCTAGCTGAGCCCCA
	*R*: 5′-ACAGAGTACTTGCGCTCAGGAG	*PR*: 5′-TGTGGACATCTCTTGGGCACT

### Chromatin immunoprecipitation (ChIP) assay

The occupancy of GAPDH in the promoter of various genes was assayed by chromatin immunoprecipitation (ChIP) assay as described previously [9]. In brief, the PHA-treated PBL were suspended in RPMI 1640 medium containing 1% formaldehyde (10^6^ cells/ml), and incubated at 37°C for 10 min. The crosslinking reaction was stopped by adding glycine to a final concentration of 0.125 M and the cells were collected by centrifugation. After washing twice with cold phosphate buffer saline (PBS) containing 10 µM PMSF and protease inhibitor cocktail (Sigma), the cell pellets were resuspended in SDS lysis solution (1% SDS, 10 mM EDTA, 50 mM Tris-HCl, pH 8.0) containing protease inhibitors, and incubated on ice for 10 min. To shear DNA, the cell lysates were sonicated on ice by 3 rounds of 10-second pulse at 7% output power in a sonicator (W-380, Heat systems-Ultrasonics Inc.). After sonication, the samples were centrifuged at 14,000 rpm for 10 min, and the supernatants were collected. To reduce non-specific background, the supernatants were diluted 10-fold with a dilution buffer (0.01% SDS, 1.1% Triton X-100, 1.2 mM EDTA, 16.7 mM Tris-HCl, pH 8.0, 167 mM NaCl) containing protease inhibitors and Protein A Agarose/Salmon Sperm DNA (Upstate/Millipore Corp.). After mixing at 4°C for 1 h, the mixtures were centrifuged at 1,000 rpm for 1 min to obtain pre-cleared supernatants. Specific antibody was then added to the pre-cleared supernatant and mixed at 4°C overnight. The antibody/protein/DNA complex was immunoprecipitated by adding Protein A Agarose/Salmon Sperm DNA with rotation at 4°C for 1 h, and pelleted by centrifugation. The immunoprecipitated complex was washed for 4 min on a rotating platform with 4 ml each of the buffers listed in the following order: (i) Low salt wash buffer (0.1% SDS, 1% Triton X-100, 2 mM EDTA, 20 mM Tris-HCl, pH 8.0, 150 mM NaCl); (ii) High salt wash buffer (0.1% SDS, 1% Triton X-100, 2 mM EDTA, 20 mM Tris-HCl, pH 8.0, 500 mM NaCl); (iii) LiCl wash buffer (250 mM LiCl, 1% NP-40, 1% sodium deoxycholate, 1 mM EDTA, 10 mM Tris-HCl, pH 8.0); and (iv) TE buffer (10 mM Tric-HCl, pH 8.0, 1 mM EDTA). After washing, the protein/DNA complex was eluted from the immunoprecipitates by incubation in 500 µl of elution buffer (1% SDS, 0.1 M NaHCO3) at room temperature for 15 min twice. To reverse the formaldehyde crosslinks of protein/DNA complex, 40 µl of 5 M NaCl was added to the combined eluate (1000 µl) and incubated at 65°C for 4 h. After incubating with protease K (40 µg/ml) at 45°C for 1 h, the DNA in the solution was extracted by phenol/chloroform and collected by ethanol precipitation. The primers used for the detection of antibody-bound chromatin DNA in the promoter of different genes were listed in Table 1.

### Immunoflurorescence confocal microscopy

Cells were fixed with 4% paraformadehyde in a slide and then permeabilized in PBS containing 0.1% Triton X-100 for 10 min at room temperature. The permeabilized cells were then incubated with a blocking solution (PBS containing 0.1% Triton X-100 and 5 mg/ml of bovine albumin) for 20 min to minimize the background staining. To detect GAPDH in the fixed cells, the rabbit anti-human GADPH antibody was added to the cells and incubated for 1 h. After washing the cells three times with PBS containing 0.1% Triton X-100, goat anti-rabbit IgG antibody conjugated with FITC was then added and incubated for another 45 min at room temperature. Finally, the cells were washed and coverslide was mounted onto the slide in Vectashield mounting medium containing DAPI (Vector Laboratories, H-100). Immunofluorescence analyses were performed with Leica TCS SP2 confocal laser scanning microscope (Leica Microsystems Inc., Germany) using 100× objective lens at 0.4 µm intervals.

## Results

### Differentially expressed proteins in the PHA-treated PBL

**Figure 1:**
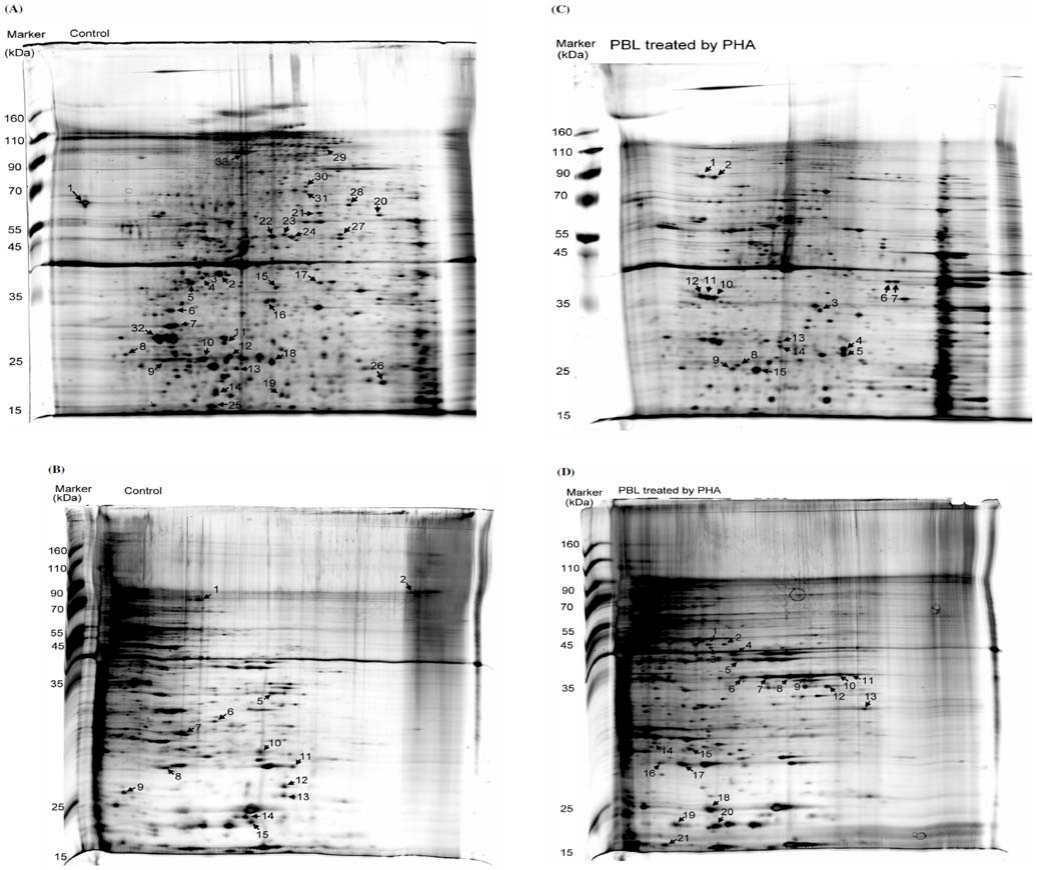
Identification of proteins that are differentially expressed in the activated T cells. Activation of T cells was done by cultivating PBL in the presence of PHA at 5 µg/ml for 1–3 days. The protein extracts from untreated controls (panels A and B) or PHA-treated cells (panels C and D) were electro-focused in a pH 4–7 (panels A and C) or in a pH 6–11 (panels B and D) IPG strip for the 1^st^ dimension and then separated in a 10% SDS-PAGE for the 2^nd^ dimension. The protein spots that displayed at least 2-fold increase or decrease were marked by arrow in panels A–D, and referred to A1-33, B1-15, C1-15, D1-21, respectively. The identities of these proteins were analyzed by MALTI-TOF and TOF/TOF, and the results were summarized in Table S1.

To investigate the proteins that are differentially expressed in activated T cells, PBL were stimulated with PHA and the proteins from the treated cells were separated by 2-DE. Representative 2-DE results of the proteins electro-focused for pI 4–7 and pI 6–11 in the 1st dimension were shown in Fig. 1. The protein spots, which displayed at least 2-fold increase or decrease from the untreated controls, were operationally defined in this study as the differentially expressed proteins. A total of 48 protein spots displayed a decrease (Fig. 1A and B), whereas a total of 36 spots displayed an increase (Fig. 1C and D) in the activated T cells. The identity of these differentially expressed proteins was analyzed by MALDI-TOF and/or TOF/TOF, and the results were summarized in Table S1.

To facilitate the analysis, the identified proteins were grouped according to their known biochemical functions: cytoskeleton, metabolism, protein destination, or others. Since many of protein spots contain peptide sequence of the same protein, a total of 25 characterized proteins were shown to decrease in the activated cells. These proteins include 10 cytoskeleton related proteins (TPMsk1, vinculin, β5-tubulin, γ-actin, talin, fibrinogen γ-A chain precursor, fibrinogen γ chain, tropomyosin TM30-pl, α-actinin 1, and cofilin), 4 protein-destination related proteins (ubiquitous tropomodulin, heat shock cognate protein 54, protein disulfide-isomerase ER60 precursor, and DnaK-type molecular chaperone), 4 metabolism-related proteins (triosephosphate isomerase, pyruvate kinase 3, glyceraldehyde-3-phosphate dehydrogenase, and carbonate dehydratase II), and 7 were unclassified (hypothetical protein AAH17450, 14-3-3 protein zeta, sorcin, RNA-binding protein regulatory subunit, Mn-superoxide dismutase, transgelin 2, hypothetical protein FLJ34571) (Table S1). On the other hand, a total of 20 proteins were characterized to increase in the activated cells. These proteins include 3 cytoskeleton-related proteins (hematopoietic lineage cell-specific protein HS1, macrophage capping protein, and cofilin), 6 protein-destination related proteins (cathepsin D, cathepsin B precursor, 26S proteasome chain p453, proteasome subunit α type 2, peptidyl-prolyl cis-trans isomerase A, and 40S ribosomal protein S12), 5 metabolism-related proteins (pyruvate dehydrogenase E1-β subunit precursor, fumarate hydratase precursor, phosphoglycerate kinase 1, fructose-bisphosphate aldolase A, and glyceraldehyde-3-phosphate dehydrogenase) and 6 unclassified proteins (B23 nucleophosmin, aryl hydrocarbon receptor-interacting protein-like 2, hnRNP protein A2, sequence 49 from Patent WO0102600, Mn-superoxide dismutase, and mitochondrial Mn-superoxide dismutase) (Table S1).

### Multiple forms of GAPDH in resting and activated T cells

**Figure 2:**
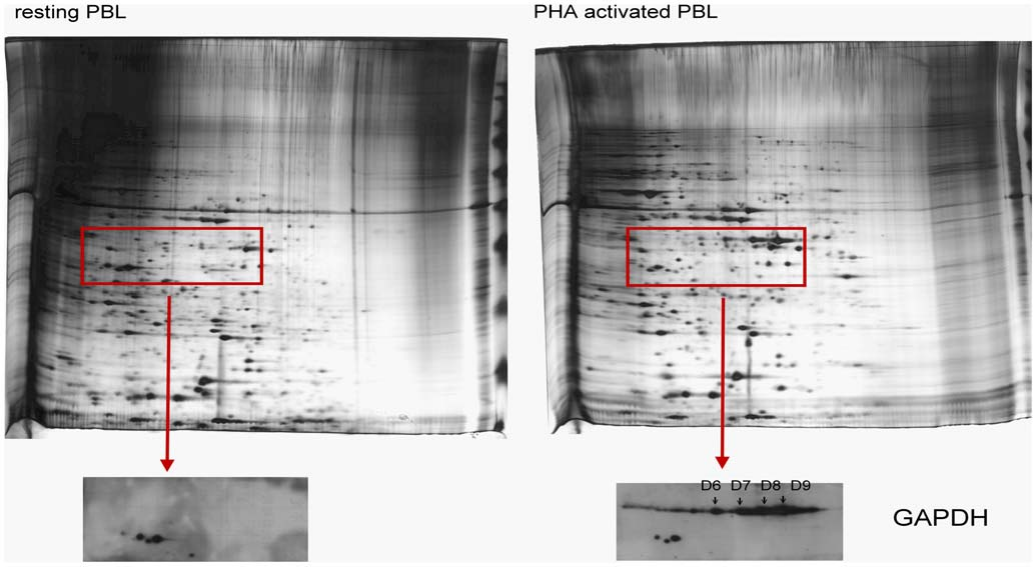
Detection of GAPDH by Western blot. The protein extracts from untreated control (left panel) or PHA-treated cells (right panel) were electro-focused in a pH 6-11 IPG strip for the 1^st^ dimension and then separated in a 10% SDS-PAGE for the 2^nd^ dimension. The area of 2-D gel containing putative GAPDH spots (boxed rectangle) was sliced out and subjected to Western blot analysis with anti-GAPDH antibody as shown in the bottom.

Among the many differentially expressed proteins identified in the activated T cells, glyceraldehyde-3-phosphate dehydrogenase (GAPDH) was unique in that it's detected in many different locations on the 2-D gels (B6, B12, B14 in Fig. 1B; D6, D7, D8 and D9 in Fig. 1D) and was detected both as an increased and decreased protein (Table S1). Since GAPDH has been thought as a housekeeping gene and is commonly used as an internal control in the RT-PCR analysis of gene expression, our identification of GAPDH as a differentially expressed protein in the activated T cells (Fig. 1B & D, and Table S1) was rather unexpected. We have confirmed that the level of GAPDH mRNA was rather constant in both unstimulated and activated T cells (7, and data not shown). Therefore, the increased GAPDH protein spots observed in the activated T cells (Fig. 1D) is likely attributed to a post-transcriptional mechanism. The molecular weight (M.W.) of protein in spots B12 and B14 are considerably smaller than the predicted M. W. of intact GAPDH and, therefore, may represent degraded forms of GAPDH. While the protein in B6, B12 and B14 spots in the resting T cells displayed a decrease in the activated T cells, the protein in spots D6, D7, D8 and D9 was increased and present only in the activated T cells. To confirm that the protein in these spots is indeed GAPDH, the area of 2-D gel containing spots B6, D6, D7, D8 and D9 was sliced (boxed rectangle in Fig. 2) and subjected to Western blot analysis by anti-GAPDH antibody. As shown in the bottom panels of Fig. 2, three low M. W. spots were detected in both resting and activated T cells, whereas the high M.W. smear containing the spots D6, D7, D8 and D9 was detected only in the PHA-activated T cells, indicating that GAPDH may exist in different modified forms in T cells.

### Cellular location of GAPDHs in activated T cells

**Figure 3:**
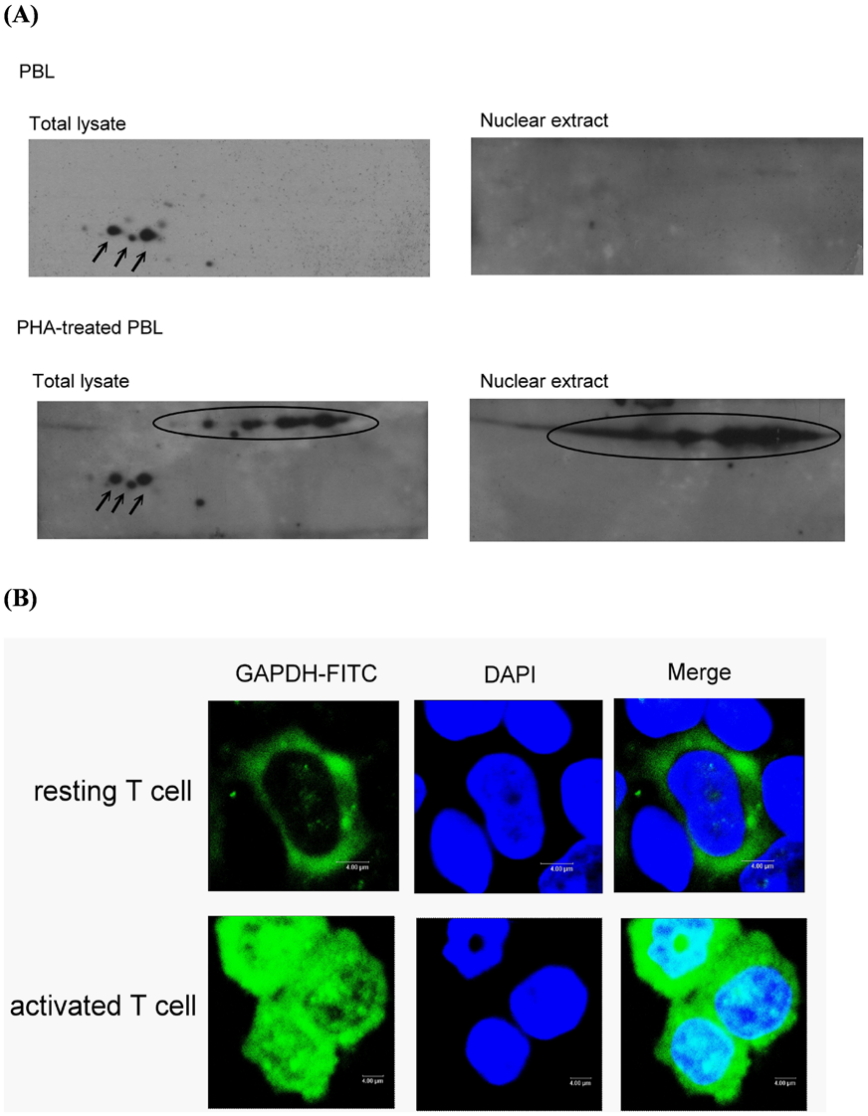
Cellular locations of GAPDHs in resting and activated T cells. (A) Total cell extract and nuclear extract were prepared from resting and PHA-activated PBL. The proteins in the extracts were separated by 2-DE, and the area of 2-D gel containing putative GAPDH spots was sliced out and subjected to Western blot analysis with anti-GAPDH antibody. (B) Resting and PHA-activated T cells were fixed and immunostained with anti-GAPDH antibody *in situ*. Immunofluorescence analyses were performed with a confocal laser scanning microscope. The location of GAPDH is indicated by green fluorescence whereas the nucleus is indicated by blue.

Although GAPDH is a key enzyme in glycolysis which is known to take place in cytoplasm, it has been shown that GAPDH has different cellular locations in non-cycling or cycling cells [10], [11]. To gain further insights about the function of modified GAPDHs in the activated T cells, the cellular locations of these modified GAPDHs were examined. The proteins from nuclear or total cell extracts were separated by 2-DE and the area of gel containing spots B6, D6, D7, D8 and D9 was sliced (boxed rectangle as shown in Fig. 2) and subjected to Western blot analysis by anti-GAPDH antibody. As shown in Fig. 3A, the three low M.W. protein spots of GAPDH (as pointed by arrows in Fig. 3A) were detected in the total cell extracts, but not in the nuclear extracts from resting and activated T cells. On the other hand, the high M.W. protein spots circled in Fig. 3A were detected in the nuclear and total cell extracts of activated T cells, but not from the resting cells. Therefore, it appears that the low M.W. GAPDHs are cytosolic and function both in the resting and activated T cells, whereas the high M.W. GAPDHs are detected only in the activated T cells. To confirm that GAPDH indeed can locate in the nucleus of activated T cells, the cellular location of GAPDHs was further examined by confocal microscopy. Resting and activated T cells were fixed by paraformaldehyde, and the distribution of GAPDH was detected by *in situ* staining with anti-GAPDH antibody. As shown in Fig. 3B, it was clear that GAPDH was present only in the cytoplasm of resting T cells, but was readily detected both in the cytoplasm and nucleus of activated T cells. While none of the resting T cells was positive for nuclear GAPDH staining, 77% and 100% of PBL activated by PHA for 24 and 48 h, respectively, was positive for nuclear GAPDH staining. Together, these results indicate that the GAPDH was post-translationally modified during T cell activation and the modified GAPDHs (protein spots D6–9 of Fig. 1D) could translocate to the nucleus of activated T cells.

### The transcriptional function of nuclear GAPDH

**Figure 4:**
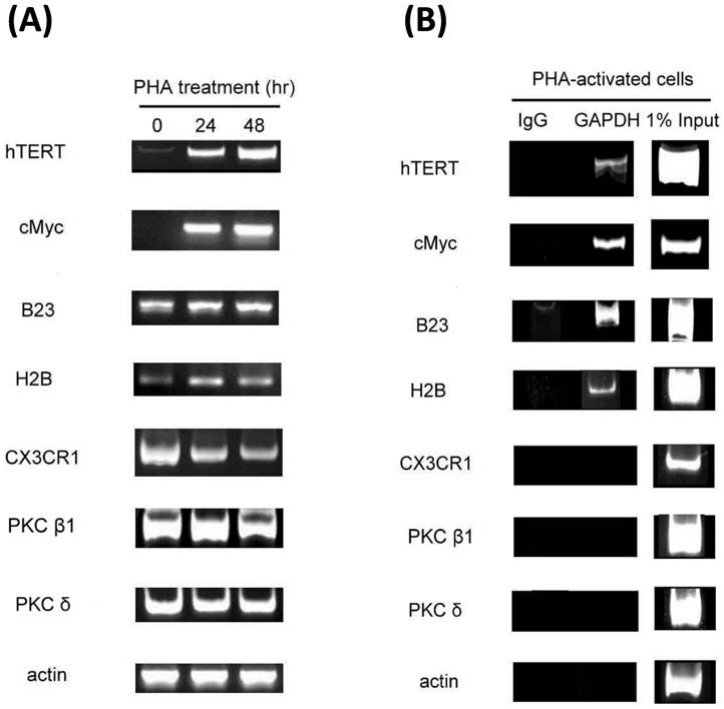
Occupancy of GAPDH on the promoter of various genes in activated T cells. (A) PBL were treated with PHA for the indicated times and the expression of genes was analyzed by RT-PCR. (B) PBL were treated with PHA for 24 h, and the promoter occupancy *in vivo* by GAPDH was assayed by ChIP. The PCR products from 1% input DNA and from DNA precipitated by anti-IgG were included for comparison.

Our detection of nuclear GAPDH in activated T cells raises the question for cellular functions of these nuclear GAPDH. In addition to its enzymatic role in glycolysis, GAPDH has been reported to participate in proliferation, apoptosis [12], [13], telomere protection [14], and transcription [11], [15]. To investigate whether the nuclear GAPDH may participate in the regulation of gene expression during T cell activation, the promoter occupancy by GAPDH was examined using chromatin immunoprecipitation (ChIP) method. The candidate genes that we selected for this study include the genes that are up-regulated (eg., hTERT, cMyc, B23 and histone H2B) [9], [15], down-regulated (eg., CX3CR1 and PKC β1) [16], or unaffected (eg., PKC δ and α−actin) [17] during T cell activation. As shown in Fig. 4A, we have confirmed, in general, that these genes were either up-regulated or down-regulated as previously reported. Interestingly, GAPDH was readily detected in the promoters of hTERT, cMyc, B23 and H2B genes, but not in the CX3CR1, PKC β1, PKC δ, or α−actin genes (Fig. 4B).

## Discussion

In this work, we have employed proteomic approach to identify the differentially expressed proteins in PHA-activated T cells. A total of 25 proteins was characterized that displayed a decreased expression, while a total of 20 proteins was characterized that displayed an increased expression in the activated T cells (). These differentially expressed proteins, in theory, may arise from altered gene expression, protein modification, degradation, or changes in protein stability. Among the proteins that displayed a decreased expression, a majority of them were cytoskeleton-related proteins, such as tropomyosin, tublin, α-actinin, talin, and viculin. These proteins are involved in the shape of cells, movement, and communication with extracellular environment through integrins [18], [19]. While the mRNA synthesis of these proteins was not examined, Western blot analysis of talin and viculin revealed multi forms of these proteins with lower molecular mass (data not shown). Decreased gene expression coupled with protein degradation is likely to account for the observed decrease of these proteins in the activated T cells.

A total of 20 proteins were characterized to increase in the activated cells. Although the increased expression of these proteins is likely resulted from transcriptional up-regulation, altered expression of protein with constant rate of mRNA synthesis has been previously observed in several studies [5], [20], [21]. We have noted that several differentially expressed proteins had multiforms with different M.W. and/or PI. Example for proteins having same M.W. but different PIs was B23 (C10, 11 and 12 in Fig. 1C). Examples for proteins having different M.W. and PIs were cofilin (B15 in Fig. 1B and D18 in Fig. 1D), hnRNP A2 (D10, 11 and 12 in Fig. 1D), and GAPDH (B6, B12, B14 in Fig. 1B and D6, 7, 8 and 9 in Fig. 1D). It is likely that these multiforms represent post-translational modification. Although not tested in this study, the different charges carried by these proteins may be caused by phosphorylation, since phosphorylated forms of these proteins have been observed and are known to modulate the protein activity [11], [22], [23].

Among the proteins that displayed an increased expression, GAPDH is unique in that it's detected in many different locations on the 2-D gels and was detected also as a protein with decreased expression. Although GAPDH is known for its key enzyme activity in glycolysis, several recent studies have revealed that GAPDH is a multifunction protein which may participate in proliferation, apoptosis [12], [13], telomere protection [14], and transcription [11], [15], suggesting that this protein may reside in the various intracellular localizations in order to perform these functions [24], [25]. Here, we demonstrated that GAPDH indeed may exist in different modified forms in T cells (Fig. 2). The three low M.W. protein spots of GAPDH (as pointed by arrows in Fig. 3A) were detected in the total lysates, but not in the nuclear extracts from resting and activated T cells, indicating that these forms are cytosolic. On the other hand, the high M.W. protein spots as circled in Fig. 3A were detected in the nucleus of activated T cells. This result is in agreement with finding that GAPDH is localized primarily in the cytoplasm of non-cycling cells, but an increased amount of GAPDH is detected in the nucleus of cycling cells [26].

To address the function of nuclear GAPDH in activated T cells, we asked whether GAPDH may participate in transcription regulation of gene expression, since GAPDH is known to regulate the expression of histone 2B (H2B) [15]. The occupancy of GAPDH in the promoter of various genes *in vivo* was analyzed by ChIP. Interestingly, GAPDH was detected in the promoters of several genes which displayed an increased expression in the activated T cells, but not in the promoters of genes that were unaffected or down-regulated (Fig. 4). Although the molecular mechanism by which GAPDH may bind to the promoter is not known at present, our finding for a preferential occupancy of GAPDH in the up-regulated genes suggests that nuclear GAPDH may participate in transcriptional regulation in the activated T cells.

## Supporting Information

Table S1 (0.11 MB DOC)
