# Supplementary material for: Proteomic Analysis of the Differential Protein Expression Reveals Nuclear GAPDH in Activated T Lymphocytes
Source: PLoS One. 2009 Jul 21;4(7):e6322. doi: 10.1371/journal.pone.0006322 (PMC2708351; doi:10.1371/journal.pone.0006322)
Supplement: Table S1 — (0.11 MB DOC) [file pone.0006322.s001.doc]

**Table S1.** Database of the differentially expressed proteins in PHA-activated T cells

| No.* | Identified protein | NCBInr | Sequence coverage | Predicted p*I* | Predicted M.W. (Da) |
| --- | --- | --- | --- | --- | --- |
| A1 | Calreticulin precursor | A37047 | 42% | 4.29 | 48283 |
| A2 | Ubiquitous tropomodulin | AAF45299 | 43% | 5.08 | 39741 |
| A4 | Hypothetical protein | AAH17450 | 38% | 5.78 | 40819 |
| A6 | TPMsk1 | AAL84569 | 29% | 4.72 | 23740 |
| A7 | TPMsk1 | AAL84569 | 50% | 4.72 | 23740 |
| A9 | 14-3-3 protein zeta, chain A | 1QJAA | 52% | 4.95 | 25069 |
| A10 | Heat shock cognate protein 54 | BAB18615 | 39% | 5.62 | 53598 |
| A11 | Protein disulfide-isomerase ER60 precursor | JC5704 | 23% | 5.98 | 57160 |
| A12 | ADP-ribosylation factor 3 | P16587 | 39% | 7.03 | 20514 |
| A14 | Viculin | AAH39174 | 15% | 5.83 | 117234 |
| A15 | α-tubulin | CAA30026 | 34% | 4.95 | 50503 |
| A16 | β5-tubulin | AAH20946 | 44% | 4.75 | 50096 |
| A17 | β5-tubulin | AAH20946 | 42% | 4.75 | 50096 |
| A18 | γ-actin | AAA51580 | 55% | 5.65 | 26147 |
| A19 | Triosephosphate isomerase | 1HTIA | 46% | 6.51 | 26807 |
| A21 | Talin | AAF27330 | 11% | 5.77 | 271653 |
| A22 | Fibrinogen γ - A chain precursor | FGHUG | 62% | 5.70 | 50092 |
| A23 | Protein disulfide-isomerase ER60 precursor | S55507 | 47% | 6.10 | 57043 |
| A24 | Fibrinogen γ chain | AAB59531 | 62% | 5.61 | 50077 |
| A25 | Sorcin | AAH11025 | 4% |  | 21947 |
| A26 | RNA-binding protein regulatory subunit | AAC12806 | 75% | 6.33 | 20050 |
| A27 | Pyruvate kinase 3, isoform 1 | AAH35198 | 26% | 7.96 | 58512 |
| A28 | Talin | AAF27330 | 13% | 5.77 | 271653 |
| A29 | Talin | AAF23322 | 11% | 5.72 | 271717 |
| A30 | Talin | AAD13152 | 7% | 5.75 | 271828 |
| A31 | Viculin | AAH39174 | 19% | 5.83 | 117234 |
| A32 | Tropomyosin TM30-pl, fibroblast | S07282 | 35% | 4.67 | 28619 |
| A33 | Viculin | AAH39174 | 36% | 5.83 | 117234 |
|  |  |  |  |  |  |
| B1 | Manganese-containing superoxide dismutase | AAP34410 | 34% | 6.87 | 23772 |
| B2 | α-actinin 1 | P12814 | 33% |  | 102974 |
| B4 | β-actin | AAH08633 | 58% | 5.56 | 41321 |
| B5 | Talin | AAF27330 | 11% | 5.77 | 271653 |
| B6 | Glyceraldehyde-3-phosphate dehydrogenase | DEHUG3 | 37% | 8.57 | 36201 |
| B7 | Carbonate dehydratase II | 1CA3 | 58% | 6.59 | 28665 |
| B8 | Transgelin 2 | AAH09357 | 78% | 8.41 | 22548 |
| B9 | DnaK-type molecular chaperone | A27077 | 22% | 5.37 | 71082 |
| B10 | Hypothetical protein FLJ34671 | BAC03786 | 37% | 9.30 | 26761 |
| B11 | Transgelin 2 | AAH09357 | 74% | 8.41 | 22548 |
| B12 | Glyceraldehyde-3-phosphate dehydrogenase | DEHUG3 | 29% | 8.57 | 36201 |
| B14 | Glyceraldehyde-3-phosphate dehydrogenase | CAA25833 | 19% | 8.26 | 36202 |
| B15 | Cofilin | S12632 | 57% | 8.22 | 18719 |
|  |  |  |  |  |  |
| C1 | Hematopoietic lineage cell-specific protein HS1 | S07633 | 24% | 4.74 | 54079 |
| C2 | Hematopoietic lineage cell-specific protein HS | S07633 | 2% | 4.74 | 54079 |
| C3 | pyruvate dehydrogenase E1-β subunit precursor | AAA60054 | 29% | 5.38 | 36807 |
| C4 | cathepsin D, chain B | 1LYW_B | 39% | 5.31 | 26457 |
| C5 | cathepsin D, chain B | 1LYW_B | 30% | 5.31 | 26457 |
| C6 | macrophage capping protein | A43358 | 33% | 5.88 | 38779 |
| C7 | macrophage capping protein | A43358 | 38% | 5.88 | 38779 |
| C8 | rho protein GDP-dissociation inhibitor 1 | I38156 | 35% | 5.02 | 23250 |
| C9 | calpain small chain | CIHUL | 30% | 5.05 | 28469 |
| C10 | B23 nucleophosmin | AAH09623 | 38% | 4.47 | 29617 |
| C11 | B23 nucleophosmin | AAH09623 | 35% | 4.47 | 29617 |
| C12 | B23 nucleophosmin | CAA34809 | 40% | 4.71 | 31090 |
| C13 | cathepsin B (EC 3.4.22.1) precursor | 1HUCB | 8% | 5.20 | 22972 |
| C15 | Rho-GDP-dissociation inhibitor Ly-GDI | A47742 | 59% | 5.10 | 23031 |
|  |  |  |  |  |  |
| D1 | Aryl hydrocarbon receptor-interacting protein-like 2 | AAM88405 | 23% | 5.71 | 36974 |
| D2 | Fumarate hydratase precursor | AAH03108 | 46% | 8.85 | 54773 |
| D3 | 26S proteasome chain p453 | S65536 | 32% | 8.23 | 45795 |
| D4 | Phosphoglycerate kinase 1 (primer recognition protein 2) (PRP 2) | P00558 | 40% | 8.30 | 44967 |
| D5 | fructose-bisphosphate aldolase (EC 4.1.2.13) | ADHUA | 62% | 8.30 | 39851 |
| D6 | glyceraldehyde-3-phosphate dehydrogenase | CAA25833 | 31% | 8.26 | 36202 |
| D7 | glyceraldehyde-3-phosphate dehydrogenase | CAA25833 | 57% | 8.26 | 36202 |
| D8 | glyceraldehyde-3-phosphate dehydrogenase | CAA25833 | 58% | 8.26 | 36202 |
| D9 | glyceraldehyde-3-phosphate dehydrogenase | DEHUG3 | 44% | 8.57 | 36201 |
| D10 | hnRNP protein A2 | AAB60650 | 51% | 8.67 | 36041 |
| D11 | hnRNP protein A2 | AAB60650 | 49% | 8.67 | 36041 |
| D12 | hnRNP protein A2 | AAB60650 | 65% | 8.67 | 36041 |
| D14 | Sequence 49 from Patent WO0102600 | CAC27316 | 64% | 7.66 | 27134 |
| D15 | Proteasome subunit α type 2 | P25787 | 59% | 7.12 | 25865 |
| D16 | Human Mn superoxide dismutase | CAA68533 | 46% | 8.66 | 24877 |
| D17 | Chain A, mitochondrial manganese superoxide dismutase | 1AP5A | 59% | 6.86 | 22288 |
| D18 | cofilin | S12632 | 48% | 8.22 | 18719 |
| D19 | Peptidyl-prolyl cis-trans isomerase A (PPIase) | P05092 | 47% | 7.82 | 18098 |
| D20 | Peptidyl-prolyl cis-trans isomerase A (PPIase) | P05092 | 65% | 7.82 | 18098 |
| D21 | 40S ribosomal protein S12 | P25398 | 35% | 6.36 | 14728 |
|  |  |  |  |  |  |

*The protein spots (A1-33 and B1-15) are decreased (＜two-fold) in the activated T cells, whereas the protein spots (C1-15 and D1-21) are increased (＞two-fold) in the activated T cells.
